# Evaluation of root surface roughness produced by hand instruments and ultrasonic scalers: An in vivo study

**DOI:** 10.34172/japid.2022.022

**Published:** 2022-11-06

**Authors:** Farzane Vaziri, Fahimeh Rashidi Maybodi, Mohammad Arab Farashahi

**Affiliations:** ^1^Periodontology Department, Faculty of Dentistry, Shahid Sadoughi University of Medical Sciences, Yazd, Iran

**Keywords:** Atomic force microscope, hand curette, piezoelectric ultrasonic, scaling, surface roughness

## Abstract

**Background.** The aim of periodontal treatment is to remove bacterial plaque and dental calculus by hand and power-driven instruments. However, the comparison of the effectiveness of these instruments has always been controversial. Therefore, this in vivo study investigated and compared the effects of hand and ultrasonic piezoelectric instruments on the roughness of dental surfaces under an atomic force microscope (AFM).

**Methods.** In this study, 35 periodontally hopeless teeth were selected and randomly divided into four groups (n=7). The control group consisted of teeth that had to be extracted for orthodontic or prosthetic treatment (n=7). In group one, scaling and root planing were performed using hand instruments. In other groups, scaling and root planing were performed using piezoelectric ultrasonic instruments with low to high power, respectively. Then the scaled teeth were extracted for analysis under an atomic force microscope.

**Results.** This study showed that root roughness significantly differed between different experimental groups (P<0.027). The root roughness (Rq) in the SRP2 group significantly differed from the control group (P<0.05), while no significant differences were observed between the other groups. Furthermore, the least roughness was observed in the SRP3 group, with the highest roughness in the SRP2 group.

**Conclusion.** Within the limitation of this study, there were no significant differences in surface roughness between different powers of the ultrasonic device.

## Introduction

 Dental plaque is the most common type of biofilm in the body.^1^* Streptococci*, especially *Streptococcus mutans*, are the most important etiologic factor in plaque formation, caries, and periodontal diseases.^2^ Extracellular glucans, especially insoluble glucose synthesized from sucrose via glucosyltransferase, cause the accumulation of native *Streptococci* on the tooth surface by binding, and together with other microorganisms in the development of extracellular polysaccharide matrix, cause the formation of dental biofilm, leading to dental plaque and halitosis.^3^ The formation of dental plaque will be visible after a few days of plaque accumulation if oral hygiene is neglected.^4^ These plaques can lead to periodontal disease, dental caries, and tooth loss if left untreated. Successful treatment of periodontal disease is not possible as long as plaque and contaminated cementum remain on the root surface.^5^

 The main goal of treating periodontitis is to remove bacterial plaque and prevent disease progression. Mechanical removal of plaque from the root surface is necessary to establish and maintain periodontal health.^6,7^ Various methods such as hand scalers, sonic and ultrasonic devices, and lasers are used for scaling and planing root surfaces to completely remove the mass microbial plaque and necrotic cement.^8^ Although the manual method, with better control of the tool and increased tactile sensation of the operator, has long been known as the standard method for scaling and planing the root surfaces, this method alone fails in some cases, resulting in periodontal disease. This method is also time-consuming and tedious and depends on individual skills.^9^

 Due to the disadvantages and problems of hand instruments, methods with more efficiency and fewer disadvantages have always been considered. Therefore, this study evaluated ultrasonic devices. This method has advantages such as access to furcations and deep grooves, less fatigue for the operator, and less treatment time.^10,11^ Choosing the right tools to remove toxins is crucial for patient comfort and reducing physicians’ fatigue. Although limitations and disadvantages are further reduced in newer and more advanced tools, their use may still have certain unavoidable consequences. Root surface roughness is one of the most fundamental changes after using precision instruments, especially in periodontal treatment^12,13^ The cumulative adverse effects of removing the particulate matter by new tools over the years may lead to severe root damage over time and facilitate the establishment of bacteria, resulting in increased plaque formation.^14^Since the rough surface with surface damage caused by scaling devices has not been studied properly in vivo and considering that a smooth surface is important to prevent plaque formation, this study evaluated root surface roughness produced by hand instruments and ultrasonic scalers in vivo.

## Methods

 The present in vivo clinical trial was performed on 35 periodontally hopeless human single-rooted incisors and premolars. All the teeth had periodontal problems, with at least 70% bone loss and Miller II mobility^14^in the examinations carried out before extraction. This study aimed to evaluate microtopography and root surface roughness using hand instruments such as Gracey curettes and low-, medium-, and high-power ultrasonic devices. This assessment was performed by an AFM device by measuring the Rq parameter. In this study, the teeth were divided into four groups by coin randomization for scaling. The first group underwent SRP using Gracey curettes 1.2, 3.4, and 6.5 (HuFriedy Co., Chicago, IL, USA). The second group underwent SRP using a low-power ultrasonic device (ultrasonic piezo scaler Uds-K, Woodpecker, China). The third group underwent SRP using a medium-power ultrasonic device (ultrasonic piezo scaler Uds-K, Woodpecker, China). The fourth group underwent SRP using a high-power ultrasonic device (ultrasonic piezo scaler Uds-K, Woodpecker, China). The fifth group, as a control, included teeth that had to be extracted due to orthodontic or prosthetic treatment plans and had healthy periodontium in the examinations carried out before extraction. The operator performed supra- and sub-gingival scaling in one session if local anesthesia was needed. For scaling, the instrument was inserted with an angulation close to 0º; then, lateral pressure was applied firmly. Scaling continued until a smooth surface was felt with a dental explorer (D&P Dental Instruments, Dena Pouya, Iran). After scaling, a groove was made in the CEJ area on both buccal and lingual sides with a high-speed diamond bur (No. 2) (Teb Bazar, Iran). The teeth were then extracted atraumatically using anterior forceps (D&P Dental Forceps, Dena Pouya, Iran) and premolars (D&P Dental Forceps, Dena Pouya, Iran). The forceps’ tip was placed in the upper area of ​​the gingival margin. The teeth were irrigated with water for 30 seconds to remove debris and blood and then immersed in a 10% formalin solution. The roughness of root surfaces (Rq) was measured through three-dimensional images of the samples with AFM-ARA (Atomic Force Microscope) (ARA AFM, Tapping Mode, Iran) with a resolution of 256×256 pixels and an N-type silicon probe with a resonance of 325 K and 125-µm cantilever length and 40-N/M contact force. In this method, both imaging (qualitative information) and analysis (quantitative information) were performed. Data were analyzed with SPSS 18, using ANOVA and post hoc Tukey tests at a significance level of 0.05. The present study has the code of ethics IR.SSU.REC.1396.2 of Shahid Sadoughi University of Medical Sciences, Yazd, Iran, with the registration code 28566 in the Clinical Trials System of Iran.

###  Result

**Figure 1:**
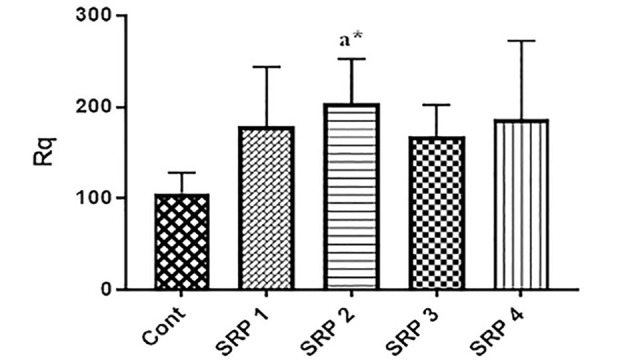
Rq index in the studied groups: Cont: control group, SRP 1: manual curette group, SRP 2: low-power ultrasonic group, SRP 3: medium-power ultrasonic group, SRP 4: high-power ultrasonic group (*P<0.05, as compared to the control).

 The mean root surface roughness (Rq) values in the control, hand curette instrument, low-power ultrasonic device, medium-power ultrasonic instrument, and high-power ultrasonic instrument groups were 105.97±22.30, 178.58±65.17, 204.44±48.13, 167.61±34.80, and 186.95±85.37, respectively (Figure 1).

 The results of ANOVA showed a significant difference in the mean Rq index between the study groups (P<0.027). Also, post hoc Tukey tests showed that the mean root surface roughness in the piezoelectric group with low power significantly differed from the control group (P=0.019). However, this difference was not significant between the other groups. The ultrasonic groups with low, medium, and high power did not significantly differ from the manual curette group. However, in using the medium power of the ultrasonic device, they showed less roughness than the manual curette group (lowest roughness). In contrast, the highest roughness was related to the low-power ultrasonic device (Table 1).

**Table 1:** The mean root surface roughness (Rq) differences in the studied groups

**Methods**	**Groups**	**Mean(Rq)**	**P-value**
**Manualcurette**	Control	7.26±29.82	0.134
	Low-power ultrasonic device	-25.86±29.82	0.907
	Medium-power ultrasonic device	10.97±29.82	0.996
	High-power ultrasonic device	-8.36±29.82	0.999
**Low-power ultrasonic device**	Control	98.47±29.82	0.019
	Medium-power ultrasonic device	36.83±29.82	0.0731
	High-power ultrasonic device	17.50±29.82	0.976
**Medium-powerultrasonic device**	Control	61.64±29.82	0.260
	High-power ultrasonic device	-19.33±29.82	0.966
**High-powerultrasonic device**	Control	80.97±29.82	0.075

**Figure 2:**
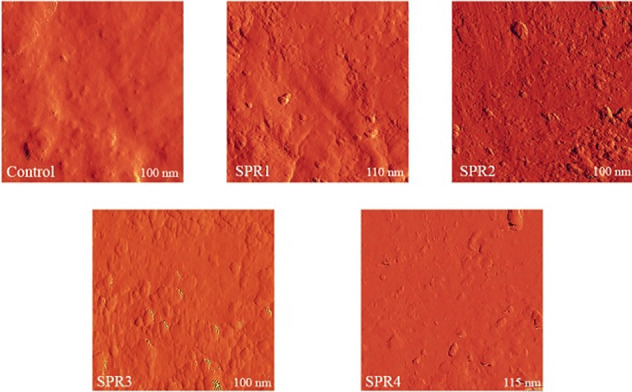
Images of AFM method; control as the control group, SPR1: hand instrument curette; SPR2: ultrasonic device with low power; SPR3: ultrasonic device with medium power; SPR3: ultrasonic device with high power.

Figure 2 shows the three-dimensional images of AFM in the control, manual curette, low-power ultrasonic, medium-power ultrasonic, and high-power ultrasonic groups. According to the AFM observations in the present study, all the methods were able to eliminate dental plaque almost completely. Despite the significant mass removal by the operator, the surface roughness of the roots was different after different interventions. The surfaces in the medium-power ultrasonic group were smoother than in the other groups and showed shallow grooves and irregularity. The roughness of root surfaces in the ultrasonic method with more or less power was more than that in the manual curette, but in the ultrasonic method, regular grooves were seen with the appearance of small islands.

## Discussion

 The present study showed that after scaling and root planing, the surface roughness (Rq) in the studied groups was significantly different between the piezoelectric group with low power compared to the control group, while the other groups were not significantly different from the control group. In general, tangible roughness was created by both hand and ultrasonic instruments. The ultrasonic groups with low, medium, and high power were not significantly different from the manual curette group. However, using an ultrasonic device with medium power resulted in less roughness than the manual curette. In this regard, the results of previous studies showed no significant differences in the tooth surface roughness after scaling between piezoelectric and manual devices in vitro.^15-17^The results of these studies are consistent with the findings of the present study, but the techniques used (profilometry and SEM) and the roughness indices of root surfaces were different from the present study.

 Previous studies have shown that piezoelectric instruments are more effective in reducing surface roughness and cause less damage to root surfaces than conventional ultrasonic devices and manual curettes.^18^ The results of studies performed using newer and thinner tips showed that piezoelectric devices can create smoother root surfaces than hand instruments.^19,20^ However, in the present study, the amount of roughness seen in the piezoelectric device group with low and high power was higher than with the manual curette, with no significant differences. This finding can be attributed to factors such as vibration method in the tool, water flow, tool tip cross-section, generator force, contact force, angle and duration of contact, tip shape, scaling endpoint, tip difference, the tool, and how to move the tip of the device to cause the amount of roughness created in these two methods. These variables and the difficulty of controlling the intervening role have made it impossible to identify^21^ the method that produces the least amount of surface roughness during the scaling procedure. Some previous studies have shown that the roughness caused by an ultrasonic device is higher than a manual instrument,^22-25^ consistent with the present study (low- and high-power ultrasonic groups) with the difference that the changes seen in the case groups of the present study were not significantly different.

 Previous studies showed a positive correlation between root surface roughness and ultrasonic device power setting: root surface roughness increased with increasing ultrasonic device power.^23,26^ However, in the present study, in general, less roughness was created with increasing power. In the medium-power ultrasonic groups, less roughness was created. Despite the use of low-, medium-, and high-power conditions in the piezoelectric device, there was no assurance that the force applied in the piezoelectric device and hand method was the same, and it seems that the force applied in piezoelectric instruments was higher than the manual method in medium power. This can be due to the operator’s tactile sense in removing roughness and the tips used. Of course, the results of different studies in this field should be compared with caution because in these studies, different methodologies and tools, such as profilometry, laser Doppler, and electron microscopy, were used, while in the present study, atomic force microscopy was used. The differences in the methods can influence the results of the studies.

 Compared to hand instruments, piezoelectric devices require less pressure and manual force, which increases the sensitivity and control of the operator during the work. Therefore, the operator can be more accurate due to the micro-vibrations of the tool’s tip. This method is safe enough because piezoelectric devices do not cut soft tissues in most cases. Also, their incision process is less invasive, causing less tissue damage, and the wound healing process is better.^27,28^ Due to the effects of cavitation in physiological solutions such as saliva and blood, the ultrasonic device creates a relatively blood-free area and increases the operator’s ability to observe the functional area compared to conventional manual techniques. Piezoelectric instruments, unlike conventional systems, are not heated, and the risk of necrosis after treatment is low.^29,30^Finally, all therapeutic procedures performed in non-surgical periodontal treatments should ultimately lead to the creation and establishment of biocompatible root surfaces for the reattachment of gingival fibroblasts.^31^The limitations of the present study included neither evaluating multi-rooted teeth nor using other methods of root surface roughness and histologic evaluation. Further studies are recommended to evaluate root surface roughness using other methods like confocal microscopy.

## Conclusion

 Within the limitations of this study, there were no statistically significant differences in surface roughness between different powers of the ultrasonic device. The piezoelectric scaler with medium power created a smoother surface than other groups.

## Acknowledgments

 The authors would like to acknowledge the Dental and Periodontal Research Center at Shahid Sadoughi University of Medical Sciences for the financial support of this study.

## Competing interests

 The authors declare no competing interests.

## Authors’ contributions

 MA initiated the research. FV and FR confirmed the design of the study. FV performed data analysis. MA and FV wrote the manuscript. All authors approved the final submission.

## Funding

 This study was supported by the Vice-chancellor for Research, Faculty of Dentistry, Shahid Sadoughi University of Medical Sciences, Yazd, Iran.

## Availability of data

 The data used to support the findings of the current study are available from the corresponding author upon reasonable request.

## Ethics approval

 The present study was approved by the Ethics Committee of Shahid Sadoughi University of Medical Sciences, Yazd, Iran (IR.SSU.REC.1396.2). The registration code in the Clinical Trials System of Iran is 28566.
